# Role of the Enterocyte in Fructose-Induced Hypertriglyceridaemia

**DOI:** 10.3390/nu9040349

**Published:** 2017-04-01

**Authors:** Simon Steenson, A. Margot Umpleby, Julie A. Lovegrove, Kim G. Jackson, Barbara A. Fielding

**Affiliations:** 1 Department of Nutritional Sciences, Faculty of Health and Medical Sciences, University of Surrey, Guildford GU2 7WG, UK; m.umpleby@surrey.ac.uk (A.M.U.); B.Fielding@surrey.ac.uk (B.A.F.); 2 Department of Food & Nutritional Sciences and Institute for Cardiovascular and Metabolic Research (ICMR), University of Reading, Reading RG6 6AP, UK; j.a.lovegrove@reading.ac.uk (J.A.L.); k.g.jackson@reading.ac.uk (K.G.J.)

**Keywords:** fructose, chylomicron, very low-density lipoprotein, triglyceride-rich lipoproteins, cardiovascular disease, de novo lipogenesis, post-prandial, apoB48, gluconeogenesis, glucagon-like peptide

## Abstract

Dietary fructose has been linked to an increased post-prandial triglyceride (TG) level; which is an established independent risk factor for cardiovascular disease. Although much research has focused on the effects of fructose consumption on liver-derived very-low density lipoprotein (VLDL); emerging evidence also suggests that fructose may raise post-prandial TG levels by affecting the metabolism of enterocytes of the small intestine. Enterocytes have become well recognised for their ability to transiently store lipids following a meal and to thus control post-prandial TG levels according to the rate of chylomicron (CM) lipoprotein synthesis and secretion. The influence of fructose consumption on several aspects of enterocyte lipid metabolism are discussed; including de novo lipogenesis; apolipoprotein B48 and CM-TG production; based on the findings of animal and human isotopic tracer studies. Methodological issues affecting the interpretation of fructose studies conducted to date are highlighted; including the accurate separation of CM and VLDL. Although the available evidence to date is limited; disruption of enterocyte lipid metabolism may make a meaningful contribution to the hypertriglyceridaemia often associated with fructose consumption.

## 1. Introduction

Cardiovascular disease (CVD) remains the primary cause of death from non-communicable diseases globally, according to the World Health Organisation [1]. An elevation of fasting or post-prandial plasma triglyceride (TG) levels are both considered as independent risk factors for the development of CVD [2]. Diet is an important modifiable risk factor for CVD, including, for example, the association of sugar-sweetened beverages with increased blood pressure, plasma TG and total cholesterol in adults [3,4]. Specifically, the consumption of the monosaccharide fructose has attracted much attention, due to reports that it raises both fasting [5,6] and post-prandial TG levels [7,8,9,10]. Several issues complicate the interpretation of these findings, such as the overall energy intake and the habitual co-ingestion of fructose with glucose in the normal diet [11]. Nonetheless, dietary fructose warrants concern, particularly with regards to post-prandial hypertriglyceridaemia (HTG), as those consuming a typical ‘Western’ diet may spend up to 18 hours per day in the fed state [12].

A raised plasma TG concentration may result from either an increased production, or an impaired clearance, of triglyceride-rich lipoproteins (TRL), and their associated remnant particles [13]. Many dietary studies in both animals and humans have investigated the effects of fructose on the metabolism of hepatic very-low density lipoprotein (VLDL), as a determinant of total plasma TG levels [14,15,16]. The liver is indeed considered to be the main organ responsible for dietary fructose metabolism [17]. However, there is also evidence that fructose-induced HTG is partly due to an increased production of intestinally-derived chylomicron (CM) particles. This has been demonstrated in a hamster model of insulin resistance [18], as well as in men with metabolic syndrome (MetS) [19]. 

The hydrolysis of CM and VLDL in the circulation by lipoprotein lipase (LPL) and hepatic lipase, results in the formation of smaller CM-remnant (CMR) particles, as well as VLDL remnants and low-density lipoprotein (LDL), respectively. Oxidized LDL particles have long been implicated in the pathogenesis of atherosclerosis [20]. However, both in vivo and in vitro evidence suggests that CMR also contribute to atherosclerotic plaque formation [21,22,23], and that this process may occur without particle oxidation [24]. 

Considering recent UK and US government recommendations to limit the consumption of free sugars to no more than 5% and 10% of total dietary calories respectively [25,26], this review will highlight the emerging evidence regarding the role of intestinal enterocyte metabolism in fructose-induced HTG, and thus CVD risk. 

## 2. Fructose Absorption and Metabolism in the Enterocyte

Fructose is typically consumed either as a free monosaccharide, or as part of the disaccharide sucrose. In the UK population, adults aged 19–64 years old habitually consume a total of 95–100 g/day of non-milk extrinsic sugars, with 40–50 g/day and 15–18 g/day specifically attributed to sucrose and fructose respectively [27]. In the United States, high fructose corn syrup (HFCS), which provides glucose and fructose as free monosaccharides (55% fructose and 42% glucose; or 42% fructose and 53% glucose) [28], accounts for a significant proportion of the 54.7 g/day of fructose consumed by all ages/genders [29,30]. Although the consumption of total added sugars in the United States decreased from 100 g/day in 1999 to 77 g/day in 2008, among children and adults < 35 years old [31], a similar trend was not observed during the same period in the UK population [32]. 

**Figure 1:**
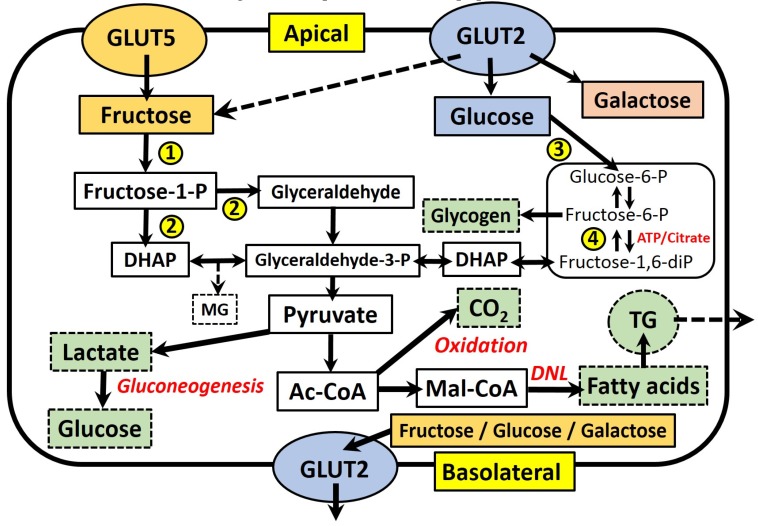
A schematic representation of the absorption and principal metabolic pathways of fructose and glucose within enterocytes. Fructose and glucose differ in their intracellular metabolism, with glycolytic enzymes subject to control by insulin, ATP and citrate, whereas the phosphorylation of fructose is not controlled. The main metabolic end products are glycogen (glycogenesis), CO_2_ (oxidation), lactate, glucose (gluconeogenesis) and fatty acids (de novo lipogenesis). Key enzymes are numbered: (**1**) fructokinase; (**2**) aldolase B; (**3**) hexokinase/glucokinase; (**4**) phosphofructokinase. Abbreviations: Ac-CoA, acetyl-CoA; DHAP, dihydroxyacetone-phosphate; DNL, de novo lipogenesis; Mal-CoA, malonyl-CoA; MG, methylglyoxal; -P/-diP, phosphate/diphosphate; TG, triglyceride.

Figure 1 summarises the absorption and main metabolic pathways of fructose and glucose within fructose-metabolising cells, which include hepatocytes, enterocytes and kidney tubular cells [17]. Fructose is absorbed across the apical membrane of intestinal epithelial cells via an energy-independent mechanism, which requires the transmembrane transporter protein GLUT5 [33]. The expression and function of GLUT5 is upregulated in response to fructose availability, leading to increased absorption of dietary fructose [34]. This process requires the intracellular metabolism of fructose in the cytosol, via the enzyme fructokinase [35]. The majority of absorbed fructose enters the circulation across the basolateral membrane of enterocytes via the related GLUT2 transporter, which also facilitates the basolateral transport of absorbed glucose and galactose. In response to high luminal concentrations of mono- and di-saccharides, GLUT2 may also translocate to the apical membrane to increase the absorptive capacity of fructose, as well as glucose and galactose [36]. Fructose that is not directly absorbed also appears to have important interactions with the non-human cells comprising the gut microbiota. Studies in rodents indicate that the negative effects of high-fructose feeding, such as MetS and oxidative stress, are correlated with changes in the gut microbial composition, including increases in Sutterella, Coprococcus and Ruminococcus bacteria, as well as a decreased abundance of Firmicutes bacteria [37,38]. Similarly, it has also been shown that fructose-induced MetS may be ameliorated by dual probiotic treatment with ***Lactobacillus curvatus*** HY7601 and ***Lactobacillus plantarum*** KY1032 [39]. Further discussion of this expansive and novel area of investigation, however, is beyond the focus of the current review. 

Within cells, fructose is rapidly converted to fructose-1-phosphate through the action of fructokinase, and subsequently to the three-carbon intermediates dihydroxyacetone phosphate (DHAP) and glyceraldehyde (GA), by the action of aldolase B. Fructose-derived carbons may then cross into the glycolysis pathway through conversion to glyceraldehyde-3-phosphate (G-3-P), making them available for energy generation through the tricarboxylic acid cycle via acetyl-CoA. Gluconeogenesis (via lactate) or glyceroneogenesis (via dihydroxyacetone phosphate) to form either glucose or glycerol, are also important. One further pathway is the conversion of acetyl-CoA, via malonyl-CoA, into new fatty acids (de novo lipogenesis, DNL). These fatty acids are esterified to form TG and either stored within intracellular lipid droplets, or packaged into lipoproteins for secretion into the circulation. The importance of DNL will be further discussed in Section 3. 

Sun et al. (2012, [40]) provided one of the best available summaries to date of the quantitative disposal routes of fructose, ingested both with and without glucose or sucrose. Based on the results of 34 stable isotope tracer studies, it was concluded that oxidation (30.5%–62%), gluconeogenesis (28.9%–54%) and lactate formation (~28%) are the most important pathways, with only a minor amount of fructose channeled towards lipid formation (<1%). The authors were unable to provide an estimate of the relative contributions of glycogen (glycogenesis) and glycerol (glyceroneogenesis) formation. It was also noted that the gender, health status and intake level of subjects impacted on their conclusions. 

While it is well established that the liver is the primary fructose metabolic organ, small intestine enterocytes are also known to express all of the necessary fructose-metabolising enzymes [41], as well as those associated with gluconeogenesis and lipogenesis (see Figure 1). In addition, their quantitative contribution to fructose metabolism remains unsubstantiated [17]. There have been few studies to accurately determine portal vein fructose concentrations in response to a fructose load, and to thus confirm the assumption that fructose is primarily delivered to the liver, rather than being subject to metabolism within the enterocyte. It is therefore possible that the relative contribution of the enterocyte to whole body fructose metabolism may be significant. Patel et al. (2015a) estimated that as much as 10%–30% of an absorbed fructose load may undergo catabolism within enterocytes, based on the results of studies in swine, guinea pigs and rats [42]. The same group reported portal fructose concentrations for mice consuming a diet containing 20% fructose (10% sucrose, 0% glucose) as ranging from ~0.06 mM during fasting to ~0.18 mM 8 h after feeding [43]. Unexpectedly, this diet also resulted in significantly higher portal glucose concentrations (~12.5–19 mM) than when feeding a 20% glucose (10% sucrose, 0% fructose) diet (~9–14 mM), which would appear to suggest significant gluconeogenesis of fructose within enterocytes. 

The subsequent sections of this review will summarise some of the key emerging evidence supporting a significant role for the enterocyte in the observed effects of fructose consumption on HTG. 

## 3. Fructose and Intestinal de novo Lipogenesis (DNL)

**Figure 2:**
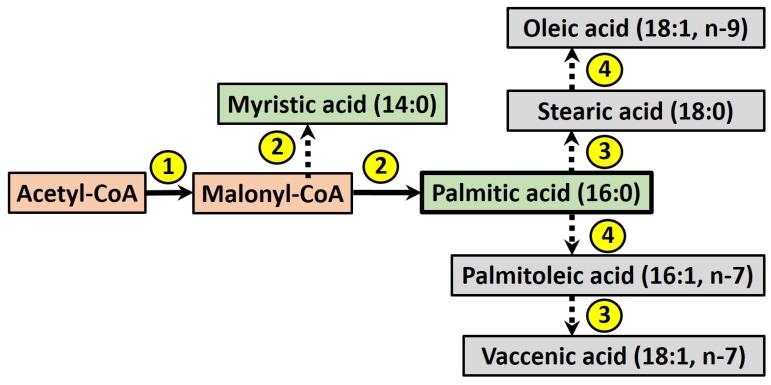
The process of de novo lipogenesis (DNL) in humans, where palmitic acid is the major product, which may be further elongated or desaturated to form other fatty acids. Enzymes are numbered: (**1**) acetyl-CoA carboxylase; (**2**) fatty acid synthase; (**3**) fatty acid elongases; (**4**) Δ9-desaturase.

The term DNL refers to the endogenous production of newly-synthesised fatty acids (FA), which may be stored within the intracellular lipid pool or secreted within lipoprotein particles. During this process (see Figure 2), acetyl-CoA is converted into malonyl-CoA by acetyl-CoA carboxylase, which is then used as a building block by the enzyme fatty acid synthase to produce myristic acid (14:0), in turn elongated to give palmitic acid (16:0). Fatty acids may be further elongated and desaturated by other enzymes [44], although palmitic acid is considered the major product of DNL in humans [45].

Differences in the metabolism of fructose, as compared to glucose, may mean it is a more potent precursor for DNL. As outlined in Figure 1, fructose only requires a single phosphorylation step via fructokinase to form fructose-1-phosphate, before its conversion to the three-carbon compounds DHAP and GA. The activity of fructokinase is not regulated, whereas the metabolism of glucose is subject to insulin-mediated expression of glycolytic enzymes, as well as the inhibition of phosphofructokinase by ATP and citrate [46]. This controls the metabolism of glucose relative to cellular energy requirements. The lack of such feedback inhibition for fructose is hypothesised to provide a large precursor pool of triose-phosphates (GA, G-3-P and DHAP) for DNL, and therefore the incorporation of newly-synthesised FA into secreted lipoproteins, consequently raising plasma TG levels. The accumulation of triose-phosphates may also favour a concomitant increase in the production of methylglyoxal (MG). This acts as a precursor for the formation of advanced glycation end products (AGEs), which have gained much attention due to their implication in the pathogenesis of several age-related diseases, including type 2 diabetes and CVD [47]. Although evidence concerning the effects of fructose-derived AGEs remains limited, it has recently been posited that they may exacerbate hepatic lipogenesis via inactivation of hepatic adenosine monophosphate-activated protein kinase [48]. A thorough discussion of the effects of MG and fructose-derived AGEs is beyond the scope of the current review, however, and readers are referred to several recent articles on the effects and potential mechanisms of AGEs [47,49,50,51,52]. 

Most attempts to quantify fructose-induced DNL in humans have assessed the appearance of de novo FA in VLDL secreted from the liver. For example, Schwarz et al. (2015) found that in healthy males the total hepatic DNL, as measured using sodium [1-^13^C]-acetate, was significantly higher (7.6%) after 9 days of a 25% fructose diet versus a complex carbohydrate control diet [53]. Chong et al. (2007) utilised a [U-^13^C]-fructose tracer to assess the amount of DNL from fructose (0.75 g/kg body weight) in 14 healthy subjects (8 male [M], 6 female [F]) and found that [^13^C]-palmitate only accounted for ~0.4% of total circulating VLDL-TG [54]. However, it was noted that this value may have been underestimated, due to inability to account for the true isotopic tracer enrichment in the precursor pool (acetyl-CoA). A larger 2.9-fold difference in [^13^C]-TRL palmitate was reported by Egli et al. (2013) during an oral fructose test (0.2 g/kg fat-free mass; 0.1% [U-^13^C]-fructose) in 8 healthy males, following a 4-day diet either high (30% of energy) or low in fructose [55]. This difference did not attain significance due an unexpectedly high inter-individual variation. Nonetheless, these studies demonstrate that the ingestion of fructose is associated with some degree of de novo lipid synthesis within hepatocytes, and that fructose can be used as a precursor. There is also limited evidence, however, suggesting fructose-simulated DNL occurs in enterocytes of the small intestine.

Haidari et al. (2002) reported DNL within primary enterocytes isolated from Syrian golden hamsters fed either a high-fructose (60%) or a chow diet for 3 weeks [18]. Enterocytes from fructose-fed animals exhibited an almost 3-fold greater incorporation of the radio-isotope label [^3^H]-acetate into FA (vs. chow-fed), indicating not only the presence of enterocyte DNL, but its upregulation in response to chronic fructose feeding. The authors also reported the incorporation of [^14^C]-fructose into TG secreted by the enterocytes, confirming the ability of fructose to act as a DNL substrate, although the labelled TG was only approximately 25% of that secreted by hepatocytes treated in the same manner. 

The capacity for intestinal DNL in humans was demonstrated by Theytaz et al. (2014) [56]. Eight healthy volunteers (4M, 4F) were given one of three mixed meals containing protein and lipid (both 0.3 g/kg body weight; ProLip), ProLip plus fructose (0.5 g/kg; Fr) or ProLip plus fructose and glucose (both 0.5 g/kg; Fr + G), with the fructose component containing 1% [U-^13^C]-fructose to trace its metabolism. Both Fr and Fr + G meals led to detectable [^13^C]-palmitate in the CM-TG fraction, indicating intestinal DNL with fructose as the substrate. The CM-[^13^C]-palmitate concentration tended to be higher with Fr + G than for the Fr meal but was non-significant, although was accompanied by significantly lower fructose oxidation and gluconeogenesis, indicating the Fr + G meal favoured intestinal DNL. A similar difference was not observed for the [^13^C]-palmitate concentration in VLDL-TG, although this was approximately three-fold greater than the CM-[^13^C]-palmitate concentration. 

Another study by the same group, utilised the same protocol (ProLip and Fr + G meals) with 8 patients who had undergone Roux-en-Y gastric bypass surgery (RYGB) and 8 control subjects. The study again demonstrated the capacity for DNL from fructose within human enterocytes [57]. In control subjects, the reported incremental area under the curve values (iAUC) for CM-[^13^C]-palmitate were almost 2-fold greater than the VLDL-[^13^C]-palmitate iAUC, suggesting enterocytes contributed more de novo palmitate to the overall circulating TRL-TG pool than hepatocytes. For RYGB subjects, there was no such difference between CM and VLDL. Interestingly, RYGB surgery appeared to have no effect on intestinal DNL capability, which is perhaps surprising, as this procedure typically bypasses the proximal (duodenum) small intestine and therefore prevents nutrient absorption from this section. This may indicate that DNL occurs more distally within enterocytes of the jejunum and ileum. However, such an observation is speculative. 

There are several limitations to both of the aforementioned human studies, as clearly acknowledged by the authors. Perhaps most importantly, lipoproteins were separated by density gradient ultracentrifugation according to their Svedberg flotation rate (S_f_), with the S_f_ > 400 fraction considered to represent predominately CM, although this may have also contained large buoyant VLDL. Similarly, the S_f_ 20-400 VLDL fraction would also have contained CMR particles. Due to the low [^13^C]-fructose content of the test meals, the [^13^C]-enrichment in the acetyl-CoA precursor pool could not be determined, and therefore the amount of intestinal DNL from fructose could not to be accurately quantified. However, as was noted, the intestinal contribution to overall DNL may have been underestimated due to the shorter half-life, and therefore higher turnover, of CM in the circulation. Thirdly, the amount of energy differed between test meals, and there was a lack of starch and fibre. In spite of these methodological issues, these novel findings demonstrate the capacity of human small intestine enterocytes to synthesise de novo FA from fructose, which may make a more meaningful contribution to post-prandial HTG than previously appreciated.

## 4. Fructose Effects on Intestinal Lipoprotein Secretion

### 4.1. Lipid Storage and CM Secretion from the Enterocyte

The small intestine was long considered to play only a passive role with regards to TG metabolism, involved solely in the absorption, re-esterification and secretion of dietary FA as TG within CM particles, thus providing lipids to the liver, adipose tissue and muscle for either storage or energy production. However, it is now recognised that transient cytosolic lipid droplets (LD) form within enterocytes following a high-fat meal, and that these dynamic organelles are an important contributor to subsequent circulating TG levels [58,59]. In the post-prandial state, absorbed dietary TG may be directly utilized for CM formation, or stored within LD to provide TG for lipoprotein synthesis during the inter-prandial period. In this way, LD formation and mobilisation allows a gradual release of absorbed dietary lipids into the circulation, regulating the post-prandial TG response [60].

A disruption of this intestinal ‘buffering’ effect on post-prandial TG levels is recognized as an important contributor to the dyslipidaemia seen in MetS and type 2 diabetes in animal models and humans, whereby fasting and post-prandial CM production is increased [61]. 

### 4.2. Fructose Effects on Apolipoprotein B48 Concentration

CM formation is a complex process unique to enterocytes, which is beyond the scope of the current review and has been extensively discussed elsewhere [62,63,64,65]. One common feature is that CM contain a single structural apolipoprotein B48 (apoB48) molecule [66], which is required for their synthesis and lipidation within the lumen of the endoplasmic reticulum (ER). Therefore, plasma apoB48 concentration serves as a measure of CM particle number (including CMR). However, it must be noted that CM are known to be very heterogeneous in size, with a diameter ranging from 75 to 1200 nm depending on the TG content [62], and may be influenced by several factors, including the amount and type of dietary fat ingested [64,67], age [68], gender [69] or genetic influences [70]. 

Chronic feeding of a high fructose (60%) diet vs. a chow diet for 2–3 weeks has been shown to induce insulin resistance and to increase both total plasma and TRL apoB48 levels in Syrian golden hamsters [18,71], which are considered to have a similar lipoprotein metabolism to humans [72]. This was shown to be due to an enhanced apoB48 stability and secretion, with a tendency towards the production of larger TG-rich CM-like particles in the S_f_ > 400 fraction [18]. 

Fructose feeding may increase apoB48 concentrations by interfering with the action of insulin, which has been shown to reduce intestinal lipoprotein secretion in insulin sensitive individuals [73]. The same fructose-fed insulin resistant (FFIR) hamster model showed a lack of insulin-mediated suppression of post-prandial TRL apoB48 synthesis and secretion, in comparison to chow-fed animals, suggesting that chronic fructose may attenuate insulin signalling [74]. This was supported by a subsequent study, where sensitisation of FFIR hamsters with rosiglitazone ameliorated TRL apoB48 over-production by 50% in cultured enterocytes [75]. Further work indicated that several aspects of the insulin signaling cascade within the enterocyte may be disrupted, including decreased insulin receptor substrate 1 (IRS-1) expression and phosphorylation, as well as an enhanced basal extracellular signal-related kinase (ERK) activity, which is associated with increased activation of sterol regulatory element binding protein (SREBP), a transcription factor known to regulate the expression of lipogenic enzymes [76]. The expression and functioning of scavenger receptor class B type I (SR-BI), a protein recognised as an important regulator of post-prandial intestinal lipoprotein production [77], is also upregulated in FFIR hamsters, and is associated with increased total apoB48 secretion [78]. 

Chronic fructose consumption may also perturb the action of the two gut hormones glucagon-like peptide 1 and 2 (GLP-1; GLP-2), which are co-secreted by intestinal endocrine L cells in response to nutrient availability in the lumen [79]. While GLP-1, which also stimulates insulin secretion, is known to inhibit intestinal lipoprotein production and therefore post-prandial HTG [80], GLP-2 has been shown to increase enterocyte fat absorption and lipoprotein output in response to a meal [81]. These opposing effects have been reported for Syrian hamsters, with a co-infusion of GLP-1 and GLP-2 yielding a net increase in lipid absorption, TRL-TG and TRL apoB48 levels in both healthy and FFIR animals. After chronic fructose-feeding there was a more pronounced increase in TRL-TG and TRL apoB48 concentrations, suggesting fructose may lead to either a decreased GLP-1 sensitivity or GLP-2 hypersensitivity [72]. A recent human study, however, failed to show such an effect in 65 healthy obese males (BMI 26.5–40.2 kg/m^2^) following a fat-rich solid meal, with only weak correlations between GLP-1 and GLP-2 AUCs and the AUCs for both CM and total TG, as well as apoB48 [82]. It is possible that altering the meals to test the specific effect of fructose in comparison to glucose and/or a complex carbohydrate control may have revealed a relationship. 

Taken together, the above Syrian golden hamster studies appear to highlight the importance of fructose-induced insulin resistance, as well as possible disruption of GLP-1 and GLP-2 signalling, as modulators of intestinal lipoprotein secretion. However, several issues such as the high-fructose content of the diets employed and the lack of comparison with either glucose or sucrose supplemented diets, make it difficult to attribute these effects specifically to fructose, as opposed to more general effects of a high sugar and/or hypercaloric diet. 

Only a few studies to date have measured the effect of fructose on apoB48 concentrations in humans. Egli et al. (2013) assessed the effect of a four-day high-fructose (HFr) diet (30% energy) versus an isocaloric control diet, where fructose was substituted for complex carbohydrates, in young (21.5 ± 2.7 years), healthy (BMI 22.1 ± 1.9 kg/m^2^) male subjects [55]. In comparison to the control diet, the HFr diet resulted in significantly higher plasma apoB48 and TRL-TG concentrations, both in the fasted state (approximately two-fold) and following an oral fructose challenge (0.2 g/kg), indicating an enhanced intestinal CM production. 

A similar study in 9 young (21.2 ± 0.3 years) healthy weight (BMI 20.5 ± 0.8 kg/m^2^) Japanese women found that the acute consumption of fructose (0.5 g/kg body weight) in combination with an oral fat tolerance test (OFTT) consisting of cream (0.35 g/kg as fat) led to a higher plasma apoB48 concentration, with a delayed peak (higher at 4 and 6 h), in comparison to either fructose, fat or an equivalent amount of glucose given alone [83]. This was also associated with significantly higher serum TG and TRL remnant-like particle (RLP)-TG. A follow-up study by the same group, again using healthy Japanese females (*n* = 12), varied the relative proportions of glucose and fructose (*w*/*w*) in the test meal (0.5 g/kg body weight), giving either 100% glucose or fructose (G100; F100), 90% fructose and 10% glucose (F90G10) or 55% fructose and 45% glucose (F55G45), in combination with the same amount of OFTT cream [84]. All drinks significantly increased plasma apoB48 levels at 2 and 4h post-prandially. However, 6h after all fructose-containing drinks, apoB48 levels remained significantly raised above fasted values, while that for G100 returned to pre-meal levels. Four hours after the F100 drink, apoB48 tended to be higher (5.2 ± 0.6 µg/mL) than for G100 (3.9 ± 0.6 µg/mL), although this difference did not attain statistical significance (*p* = 0.09), perhaps owing to the relatively small sample size, as commented on by the authors. The RLP-TG response followed the same pattern as for apoB48. Taken together, these observations may suggest either a greater CM secretion, or a delayed plasma clearance, in response to fructose. 

The aforementioned study by Theytaz et al. [56] found that apoB48 iAUC values following the Fr and Fr + G meals tended to be higher than for ProLip, although this difference was not significant. It is possible that these differences may have proved significant with a higher number of participants and thus greater statistical power [56].

Xiao and colleagues (2013) conducted one of the most rigorous investigations to date on the effects of fructose on apoB48 concentrations [85]. Seven healthy males, without impaired lipid metabolism, received an intraduodenal infusion of a 20% Intralipid (IL) solution (60 mL/h) over a 14 h period, either with or without a co-infusion of glucose (IL + G) or fructose (IL + Fr) solution (both 20%, 60 mL/h) and under conditions of an intravenous pancreatic clamp, to control for the effects of feeding on insulin, somatostatin and growth hormone. A primed constant infusion of [^2^H_3]_-leucine (10 µmol/kg bolus plus 10 µmol/kg/h for 10 h) was started 5 h into the study in order to measure apoB100 and apoB48 kinetics in the fed state. TRL-apoB48 production rate was significantly increased during both the IL + G (*p* < 0.01) and IL + Fr (*p* < 0.05) infusions (versus IL). The IL + Fr infusion also significantly raised TRL-apoB100 production rate (PR; *p* < 0.05). Although IL + G infusion led to a corresponding increase in the TRL-apoB48 fractional clearance rate (FCR; *p* < 0.05), in the IL + Fr group such an effect on FCR was not observed. These effects on apoB lipoprotein kinetics were accompanied by an increase in total plasma TG and TRL-TG in IL + G (*p* < 0.05 for both) and IL + Fr (*p* < 0.01 for both) groups. 

Overall, these findings clearly demonstrate that fructose and glucose have differential effects on intestinal lipoprotein particle production and their subsequent metabolism. While TRL-apoB48 concentrations were significantly raised by both IL + G (3.06 ± 0.89 mg/L vs. 1.41 ± 0.51 mg/L saline) and IL + Fr (2.14 ± 0.50 mg/L vs. 1.39 ± 0.34 mg/L saline), with fructose this was attributed solely to an increased TRL-apoB48 PR and not due to a reduction in FCR. The reason for these differences could not be fully explained by the authors, although the lack of FCR stimulation following fructose was not due to apolipoprotein C-III inhibition of LPL, as this was not significantly affected by fructose feeding. It was suggested that glucose may chemically alter the composition of TRL particles so as to favour their metabolism and/or clearance from the circulation, as the VLDL-TG hydrolysis rate from fructose-fed, as compared to glucose-fed (10% drinking solution) rats, has been shown to be impaired, as well as the removal of VLDL-TG by the liver [86]. Whether this may explain the observed differences in apoB48 kinetics remains to be seen.

Both the animal and human studies mentioned indicate that fructose increases circulating CM concentrations, as assessed by their apoB48 content. The specific mechanisms by which fructose may differentially affect CM particle production clearly require further investigation, although a disruption of insulin and/or GLP-1 and GLP-2 signalling are potential candidates, as well as effects on the composition and thus the metabolism of CM particles by the liver and other tissues possessing LPL activity (e.g., adipose tissue). 

### 4.3. Fructose Effects on Enterocyte CM-TG Secretion

There are very few human studies that have measured CM-TG secretion rates, or even CM-TG concentrations, because of analytical issues. As previously mentioned, the isolation of CM particles is typically achieved by density gradient ultracentrifugation, with an assumption that CM-TG corresponds to the S_f_ > 400 lipoprotein fraction. In fact, significant amounts of intestinally-derived TG have been shown to reside in the S_f_ 60–400 fraction (VLDL1 fraction), particularly in response to ingestion of saturated FA and during the later post-prandial phase, indicating the production of smaller, denser CM particles [87,88,89]. The only currently reported method by which TG of intestinal (CM) and hepatic origin (VLDL) may be definitively separated is through the use of density gradient ultracentrifugation combined with immunoaffinity chromatography. Monoclonal antibodies with specificity for apoB100 are used, which do not cross-react with apoB48, giving purified unbound (apoB48) CM and bound (apoB100) VLDL fractions [90]. Therefore, the separation of the CM-TG component within the S_f_ > 60 fraction (TRL) using immunoaffinity chromatography provides a greater specificity when assessing the enterocyte contribution to circulating TG levels. Sun et al. (2013) used this technique to develop a novel stable isotope protocol to enable CM-TG production to be measured in humans in vivo [91]. Shojaee-Moradie et al. (2013) [19] utilised the protocol to demonstrate an increased CM-TG concentration in men with metabolic syndrome, compared to lean individuals, which was due to an enhanced CM-TG production rate, with no difference in the CM-TG clearance rate.

The aforementioned studies of Theytaz et al. (2014) [56] and Surowska et al. (2016) [57] provide some of the only available results to date regarding the effect of fructose on CM-TG levels, although they did not isolate specific CM and VLDL fractions as described above, but used the concentration of TG in the Sf > 400 fraction as a marker of CM-TG [56,57]. In the latter study, control subjects (no RYGB surgery) that consumed Fr + G in addition to ProLip tended to show an increased Sf > 400-TG response, with a larger delayed peak and higher iAUC over the 360 min time course, although these differences were not significant, perhaps owing to the low statistical power of the study and high inter-individual variation. In the first study, providing ProLip with either fructose or fructose and glucose did not affect the CM-TG response, giving equivocal iAUC values. Interestingly, although the Fr + G meal provided a higher number of calories (7.99 ± 0.14 kcal/kg body weight) than the Fr meal (5.99 ± 0.09 kcal/kg), the lack of a significant difference in total and S_f_ > 400-TG may suggest that fructose is a more important determinant of intestinal TG secretion than glucose, and that the effects of fructose may be irrespective of overall energy intake. However, the lack of an additional ProLip plus glucose group in this study does not allow such an observation to be confirmed. Further studies are clearly required, ideally comparing equimolar and energy-balanced glucose and fructose test meals, to determine whether fructose significantly modulates CM-TG secretion. 

## 5. Conclusions

The importance of intestinal lipid metabolism has gained widespread appreciation in recent years, including the role of enterocyte LD and CM particle over-production as determinants of HTG, which may impact on CVD risk. Controversy persists concerning the deleterious effects of fructose consumption on human health [11,92,93], mainly due to its habitual co-ingestion with glucose in the normal diet (e.g., HFCS and sucrose) and potential confounding effects of hypercaloric and high percent fructose experimental diets. Nonetheless, human studies addressing these key issues have still reported that fructose leads to fasting and post-prandial HTG [5,9,10,55]. Equally, it has recently been shown that restricting the fructose content of the diet (isocaloric substitution with starch) has beneficial effects in children with obesity and MetS, including reductions in fasted TG, total apoB, apoC-III and LDL cholesterol levels, as well as an increase in LDL particle size [94,95]. 

There are several putative mechanisms by which fructose consumption may contribute to HTG through disruption of enterocyte TG metabolism. Emerging evidence from rodents and humans indicates that enterocytes may make a more meaningful contribution to fructose metabolism than previously thought [42,57]. This appears to include disposal of fructose carbons via DNL, providing de novo FA to the circulation. Specific consumption of fructose may also enhance CM secretion, acting through an attenuation of the endogenous control of lipoprotein secretion by insulin, GLP-1 and GLP-2. Although evidence supporting these effects is at present in its infancy, future investigation may reveal dysregulation of intestinal lipid metabolism as an important factor in the observed effects of fructose on post-prandial TG. 

Ideally, future studies should compare the long-term effects of modest, biologically relevant amounts of fructose, as typically consumed in the habitual diet, in combination with sucrose, glucose and/or starch, and as part of an isocaloric weight-maintenance diet. It would also be useful to vary the relative proportions of fructose and other sugars administered, as together this would address the confounding issue of differential fructose metabolic handling in the presence/absence of other simple and complex carbohydrate sources (e.g., the influence of co-ingestion with glucose). Studies should also be conducted in both healthy individuals as well as in those at an increased risk of CVD, such as subjects with MetS and type 2 diabetes. Where possible, quantitative stable isotope methodology should be employed to specifically trace the metabolism of fructose, as compared to other sugars. Also, the accurate separation of CM and VLDL within isolated TRL fractions based on their specific apoB content, would enable confirmation of the relative contribution of enterocytes to fructose-induced HTG. If this is found to be significant, it may provide further evidence to support the recent UK (5%) and US (10%) public health recommendations to restrict the amount of dietary calories obtained from free sugars [25,26] for cardiometabolic disease prevention.
